# Therapeutic inhibition of RAS in non-small cell lung cancer

**DOI:** 10.3389/fonc.2026.1817943

**Published:** 2026-06-30

**Authors:** Katherine B. Nichols, Benjamin O. Herzberg

**Affiliations:** 1 Division of Hematology/Oncology, Department of Medicine, Columbia University Irving Medical Center, New York, NY, United States; 2 Herbert Irving Comprehensive Cancer Center, Columbia University, New York, NY, United States

**Keywords:** drug development, *KRAS*, lung adenocarcinoma, NSCLC, *RAS*

## Abstract

Oncogenic KRAS mutations are the most common driver event in NSCLC, occurring in up to 30% of patients. Direct strategies to target KRAS (and other RAS isoforms) met with consistent failure until the discovery of covalent KRAS G12C inhibitors, leading to a first-in-class FDA accelerated approval for the treatment of NSCLC in 2021. Since then, multiple chemotypes have been reported to extend direct KRAS targeting beyond G12C alleles, with several dozen molecules entering early or late clinical testing in rapid succession. Here, we review such strategies. We recap the chemistry and clinical strategy behind G12C inhibitors as a first step in direct KRAS targeting. We then propose an overarching classification schema for novel KRAS targeting molecules, focusing on the chemical strategies encoded in their use, and moving beyond conventional inhibitors to glues, degraders, and other novel pharmacologies. We evaluate the non-G12C, allele-specific molecules (such as those targeting G12D), inhibitors of multiple KRAS alleles (“pan-KRAS”) and inhibitors of all RAS isoforms (“pan-RAS”). We consider these molecules’ potential as monotherapy, in combination with one another, and in combination with conventional (FDA-approved) or novel (unapproved) treatments. Finally, we discuss what the future may hold for KRAS directed strategies in NSCLC.

## Introduction

Over four decades ago, *RAS* genes were identified in cancer-causing rat retroviruses (1). A few years later, single-site substitutions in *HRAS* or *KRAS* were shown to be necessary and often sufficient for oncogenic transformation (2, 3). At the time, this experimental result suggested that transformative therapies for cancer could be near at hand – what could be simpler than addressing the output of one gene, with one oncogenic substitution, to treat cancer? (4, 5) The answer to that question, it turned out, was forty or so years of qualified failure to develop strategies to directly target RAS-mutated cancers. And yet in just the last five years, direct *RAS*-targeted therapies have entered the clinic, treated patients, obtained regulatory approval, and spawned a vast race to spread and optimize their benefits. This transformation was enabled above all by pharmacologic advances which cracked open the previously undruggable RAS proteins. By serendipity, these advances first met success in non small-cell lung cancer (NSCLC), where the mutant KRAS G12C protein, which proved critical to the chemical strategy, is among the most prevalent oncogenic drivers (6). Since the first clinically successful strategy directly targeting KRAS was published in 2021 (7), dozens of direct-targeting KRAS molecules have entered clinical testing in lung, pancreatic, colorectal, and other cancers, now even moving into strategies targeting not just KRAS, but all RAS isoforms (8–10).

In setting out to review this field, we face several challenges. The first is the rapidity with which drug development for RAS is moving. We can only capture a single snapshot in time. This snapshot may be incomplete in the time between when we write and publish – never mind a year from now. To counteract this, in this review, we have focused not just on the list of currently available therapies and ongoing trials, but have attempted to both offer an overarching classification schema which will be useful to researchers and clinicians regardless of results of the coming years, and to offer our views, as clinical trialists, on the directions the field is heading. The second challenge is one of organization and review structure. Drug development is rarely a linear process. For RAS inhibition this has been especially true. G12C inhibitors are structurally related to some G12D inhibitors and some pan-KRAS inhibitors – but to others, not. These novel drugs entered the clinic at different times and with different clinical development structures. There is a tension between structuring a review as a purely clinical story – which drug is where in development – and a chemical one – which drug is related to another in the lab. We have tried to split the difference. Molecules such as G12C inhibitors, which are relatively mature, are discussed together, even as newer chemical approaches are mentioned. After this we try to group by allele, but we also split out the inhibitors of the same allele (say G12D) but different chemical genesis (molecular glue inhibitors) together with their progenitors. While this necessitates some jumps, this mixed approach allows for comparisons across similar molecules as well as across categories.

Finally, we have chosen in particular to use the term “RAS” drug development rather than exclusively “KRAS” drug development. The simplest reason is that we now have molecules which inhibit all forms of RAS – “pan-RAS inhibitors” – and molecules which inhibit only KRAS – “pan-KRAS inhibitors.” We cover the emergence of such RAS-directed strategies in this review. In addition, we believe that future directions of drug development in NSCLC and other cancers are likely to involve a combination of these molecules and others where these distinctions are key. We have tried to use these terms predictably, but for clinicians who are used to simply “KRAS inhibitors,” we recognize that this can be jarring.

## The biology and epidemiology of KRAS-mutated NSCLC

Of the three paralogous human RAS proteins, KRAS (the Kirsten rat sarcoma viral oncogene homolog) dominates the driver landscape of NSCLC. Approximately 25-30% of lung adenocarcinoma harbors a *KRAS* mutation, and alterations in *KRAS* are also observed in squamous cell carcinomas (SCC) and mixed adenosquamous tumors at rates of 4-6% (6, 11–14). *HRAS* and *NRAS* mutations are also rare but occasional drivers in NSCLC (15). KRAS is a conserved membrane-associated GTPase which activates downstream signaling cascades in both the mitogen-activated protein kinase (MAPK) and phosphoinositide 3-kinase (PI3K) pathways (and other growth pathways as well) (16–18). When bound to guanosine triphosphate (GTP), KRAS is in its active, signal-transducing state, often indicated as KRAS(GTP) or KRAS(ON). Intrinsic, slow GTPase activity in RAS converts GTP to GDP; when GDP is bound, signaling is inactive, hence RAS contains an intrinsic negative feedback loop (19). This feedback loop is modified by two classes of RAS-associated proteins: Guanine exchange factors (GEFs) switch out GDP for GTP, leading to activation; while GTPase-activating proteins (GAPs) accelerate the inherently slow GTPase activity of RAS by itself (20–22). Mutations in KRAS, which are overwhelmingly point mutations found in codon 12, 13, or 61, act by abrogating the GTPase activity of the protein and preventing GAP participation, shifting the equilibrium toward the active, GTP-bound RAS state and inducing perpetually active growth signaling. Despite this equilibrium shift, cycling between “on” and “off” complexes can still occur, and even be more frequent, in mutated KRAS, depending on the exact mutant allele (19, 23, 24).

The distribution of RAS mutations varies by cancer (25). In NSCLC, the most common mutation is KRAS p.G12C, found in 10-15% of all patients and accounting for slightly less than half of all KRAS mutations (6, 13, 26). The causal G12C DNA transversion mutation (c.34G>T) is heavily associated with cigarette smoking, accounting for both the increased prevalence of KRAS mutations in lung cancer and its overrepresentation among lung cancer patients who smoked (27, 28). By contrast, G12C is found only rarely in other tumor types which commonly harbor KRAS mutations, such as pancreatic adenocarcinoma (PDAC) or colorectal cancer (CRC) (25). Smoking signatures are, at least by comparison to lung cancers, uncommon in the genomes of those cancers (29). The association with smoking is also likely one of the main reasons for geographic and sex-based differences in the prevalence of various KRAS mutations (30–32). For example, G12D is more commonly found in never smokers compared to G12C being found universally among smokers (33). The balance of mutated alleles in lung cancer are G12V (10-20% of KRAS), G12D (10-15%), G12A (5-10%), and then other alleles (together about 15-20%) (25, 27, 28, 33–35). The distribution of these alleles may vary based on geography, with G12C more prevalent in Western populations compared to Asian cohorts (36). These differences may have practical implications for the design of clinical trials in different regions, as they do for EGFR-mutated NSCLC. In absolute prevalence terms in Western populations, KRAS G12D, present as a driver in 3-5% of all lung cancers, is about as common as ALK rearrangements, EGFR exon 20 insertions, or Met exon 14 skipping alterations (37–40). As more allele-specific inhibitors enter clinical testing beyond G12C, the regulatory pathway for rarer mutants is likely to become an important question and this allele distribution may take on outsized importance.

KRAS mutations may confer a worse prognosis, but the evidence is mixed and has shifted over time as immunotherapy and chemoimmunotherapy have entered into widespread use (6, 41–46). Studies regarding prognosis have had varying comparator arms (non-KRAS versus KRAS; differing alleles within KRAS mutants; KRAS mutated versus others but excluding EGFR/ALK/etc), making definitive conclusions difficult – worse prognosis compared to whom? PD-L1 tumor proportion scores (TPS), tumor mutation burden (TMB) measured by tissue or blood (47), smoking status, and other clinical features may have independent or dependent prognostic information when considered along with KRAS mutations. The picture is further muddied by the fact that other strong (and negative) prognostic commutations are commonly found in KRAS mutated tumors, in genes such as *STK11, KEAP1, SMARCA4* and *CDKN2A (*48, 49*).* These mutations clearly modulate prognostic effects and responses to therapy, are overrepresented in KRAS mutated lung cancer, and drive large prognosis decrements (6, 49–53). Whether a cooperativity with KRAS mutation drives these effects or there is fully independent biological action is not yet fully understood.

What is clearest about the prognosis of patients with KRAS mutant tumors is what they share with all patients with NSCLCs: lung cancer remains the number one killer of cancer patients in absolute terms (54). Outside the real but true minority who achieve cure through immunotherapy (53), most patients run out of therapies that work, rather than have too few. The search for non-chemotherapy, non-immunotherapy based strategies for RAS eventually found initial success with the discovery of G12C-specific molecules.

## Classifying RAS inhibitors

### The discovery of KRAS G12C inhibitors

The key breakthrough in directly targeting KRAS grew out of a rational application of covalency to small molecule drug discovery (55). The G12C mutation replaces an essentially inert glycine with a nucleophilic cysteine. Screening cysteine-reactive scaffolds led to the discovery of a cryptic pocket in KRAS(G12C)-GDP to which compounds could effectively bind (7). Binding to what is now termed the switch II pocket prevents the KRAS(GDP) complex from recycling to the GTP-bound form, and elevated GTP hydrolysis rates of the G12C mutant protein result in frequent GDP-GTP cycling despite a shifted equilibrium toward the on-state (56). This combination of features leads to rapid, though not instantaneous, downregulation of MAPK signaling in the presence of inhibitor which can only bind KRAS G12C(GDP). The key features of this approach are (1) a traditional small molecule inhibitor as the scaffold; (2) chemical covalency responsible for at least some of the on-target potency; (3) state specificity, against only the GDP-bound form of the protein; and (4) allele specificity, with minimal activity against non-G12C KRAS alleles or wildtype (WT) KRAS (56, 57).

### Classifying RAS inhibitors: G12C and beyond

**Figure 1:**
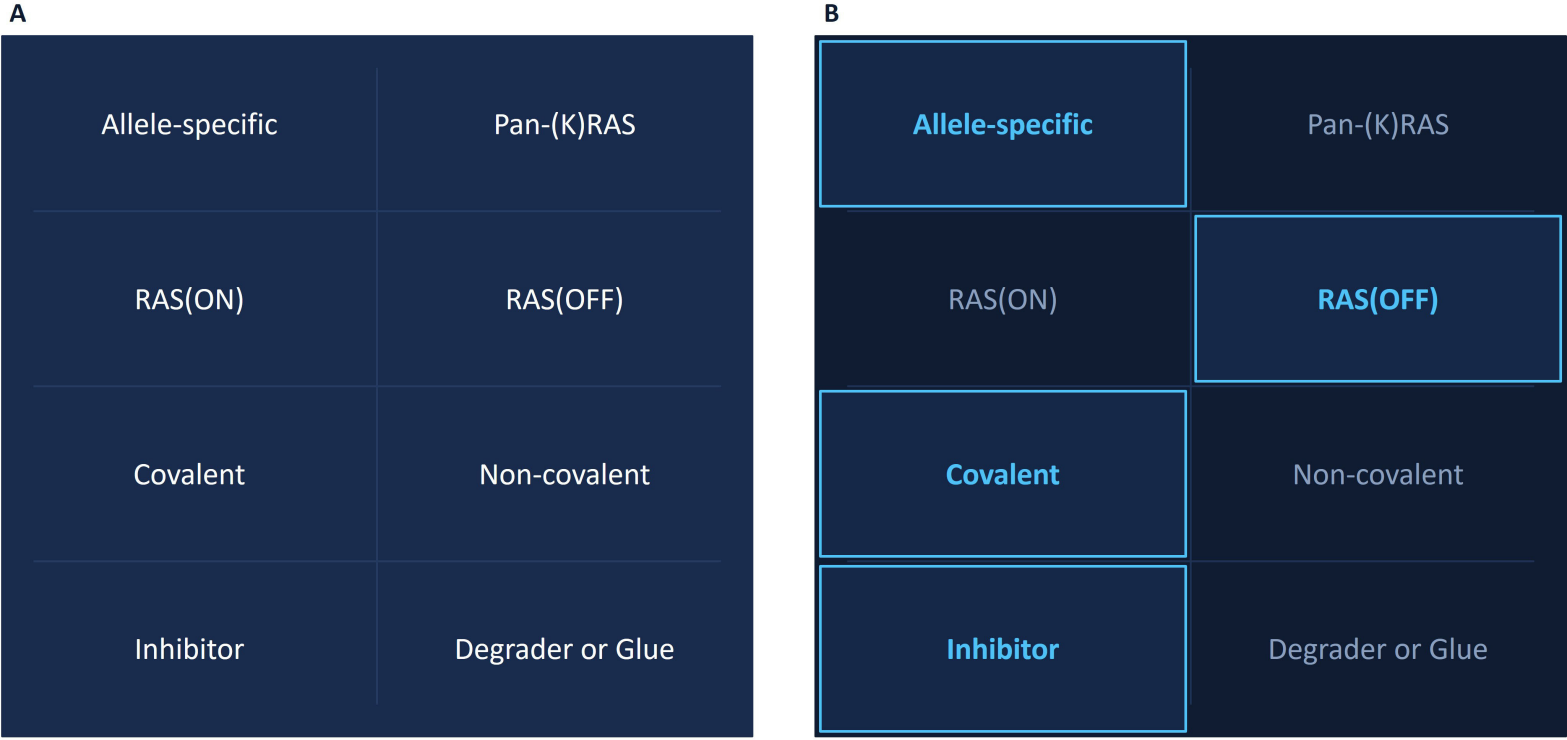
A classification schema for RAS inhibitors. Four mechanistic axes classify direct RAS-targeting agents. Allele specificity versus pan-(K)RAS coverage; covalency versus non-covalent; nucleotide-state preference for RAS(ON/GTP) or RAS(OFF/GTP), and modality (glue/degrader versus inhibitor). This dichotomous schema is laid out in **(A)**. In **(B)**, this schema is applied to the most familiar KRAS-targeting agent, sotorasib (or adagrasib).

**Figure 2:**
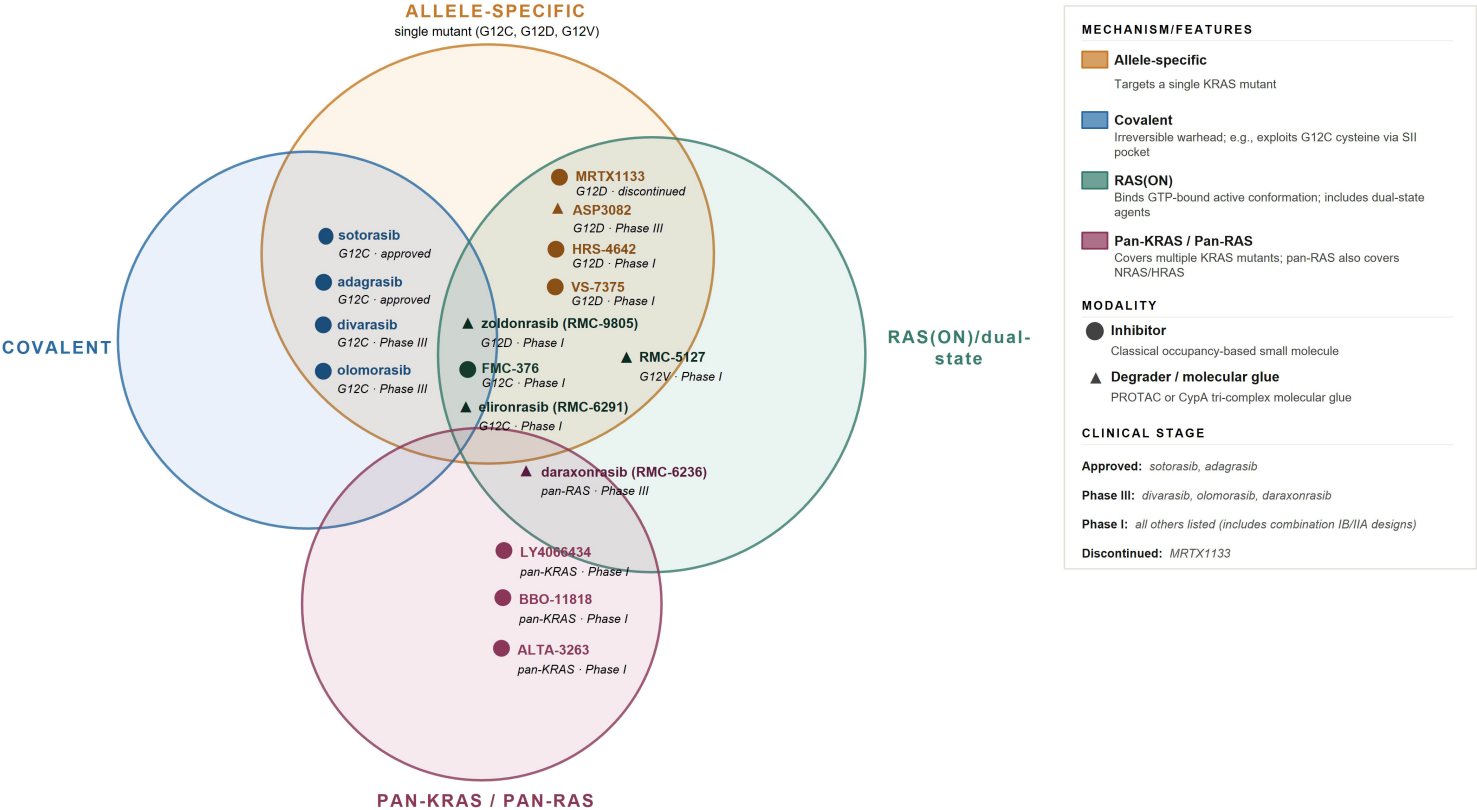
RAS therapies by classification. KRAS inhibitors laid out across classification schema. Symbol shape distinguishes modality: circles (•) indicate classical occupancy-based inhibitors; triangles (▲) indicate degraders (PROTACs, catalytic proteasomal elimination) or molecular glues (tri-complex agents recruiting cyclophilin A for steric effector blockade). Sotorasib and adagrasib (allele-specific × covalent, RAS-OFF) are the first-generation molecules. Daraxonrasib (pan-RAS × RAS-ON, tri-complex ▲) illustrates the pan- and dual-state frontier. Degraders such as ASP3082 (G12D-selective PROTAC ▲) retain their allele-scope classification while employing a fundamentally distinct elimination mechanism. Some molecules, especially the pan-KRAS inhibitors, may have some combination of nucleotide-state specificity, rather than pure (ON) or (OFF) activity.

**Figure 3:**
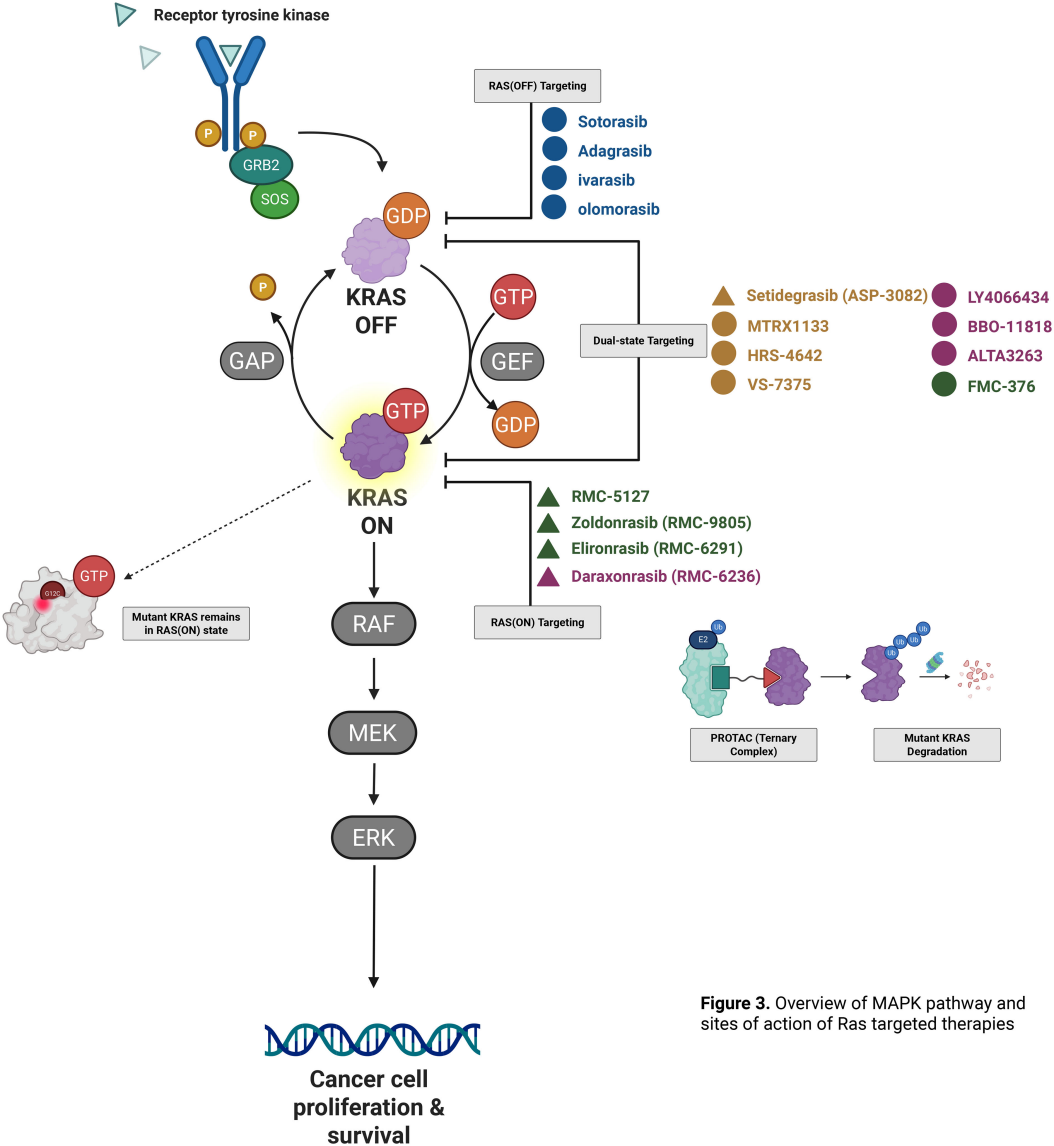
Overview of MAPK pathway and sites of action of RAS-targeted therapies. Created in Biorender, publication license: https://BioRender.com/csnm0uy.

Focusing on these features, it is worth pausing to note that we can generate a broadly useful classification schema for direct KRAS inhibitors from them (**Figure 1A**). In this schema, molecules that inhibit RAS can be direct inhibitors or multi-complex inducers, such as molecular glues or degraders; they can be covalent or non-covalent; they can act against one (invariably mutant) allele, or against mutant and non-mutant (K)RAS with similar potency; and they can bind only the GDP-bound form, the GTP-bound form, or both. This schema applied to the first generation of KRAS G12C inhibitors is also shown (**Figure 1B**) and visualized for many current RAS-targeted programs in **Figure 2**. Overlaid on a pathway schematic, these drugs are displayed in **Figure 3**.

While no simple classification can capture the full biochemical and structural complexity of RAS signaling and its pharmacologic modulation, we believe such a framework is necessary given the rapid expansion and heterogeneity of the field. Existing approaches to organizing RAS-targeted therapies have generally focused on either clinical stage of development or individual molecular mechanisms in isolation. However, these approaches can make it difficult to compare agents across trials, anticipate mechanisms of resistance, or rationally design combination strategies.

By contrast, our schema is structured around a small set of pharmacologically and biologically meaningful axes — direct versus multicomplex engagement, covalent versus non-covalent binding, allele specificity, and nucleotide-state selectivity — that together determine how a molecule interacts with RAS and perturbs signaling output. These features are directly relevant to clinical behavior: for example, allele specificity and state selectivity may influence therapeutic index, toxicity, and resistance patterns, while multicomplex engagement may enable activity in contexts where conventional binding pockets are absent. For clinicians, this schema provides a simplified way to interpret an increasingly complex therapeutic landscape, linking molecular mechanism to expected patterns of efficacy, resistance, and toxicity, and thereby supporting more informed trial selection and, perhaps one day, treatment decisions.

We therefore propose this schema not as a definitive taxonomy, but as a pragmatic framework to support cross-trial comparisons, hypothesis generation for combination therapies, and interpretation of emerging clinical data. At the same time, it has clear limitations. It does not explicitly incorporate pharmacokinetic properties, tissue distribution, or tumor-intrinsic factors such as co-mutation status, all of which are critical determinants of clinical efficacy. Some molecules may not fit perfectly within it. As the field evolves, additional dimensions — including biomarkers of response and resistance — may need to be integrated to refine this framework further.

## KRAS G12C inhibition: at the cusp of clinical maturity

### Clinical features of G12C inhibition: many molecules, modest benefits

From the first preclinical reports of potency, G12C inhibitors (G12Ci) sotorasib and adagrasib raced toward a first FDA approval in 2021 for KRAS G12C mutated NSCLC that was refractory to chemotherapy and immunotherapy (58, 59). In the years since, these molecules have become well-worn parts of the clinical armory against NSCLC in the metastatic, chemoimmunotherapy-refractory setting. This path to clinical approval for KRAS G12C inhibitors as monotherapy, and the mechanisms of response and resistance to KRAS G12C inhibition, have been reviewed in good detail elsewhere (8, 9). For the purposes of this review, we will dwell on two key aspects we have not seen previously discussed: how the field has surprised in the intervening years since the first approvals, and what are the key questions, today, surrounding the future of G12C inhibition in NSCLC. Both of these topics have broad implications for the future development of KRAS inhibitors in NSCLC beyond G12C.

Within a few years of the first report of sotorasib, between 5–10 G12C inhibitors targeting the Switch II pocket of the GDP-bound form entered clinical development and proceeded to registrational clinical trials in the United States, Europe, and Asia (8, 9). Most of these molecules reported response rates in single arm studies in the 35-55% range, with a median duration of response between 8–10 months and a corresponding progression free survival of roughly 6 months (58–61). As is usual for molecularly-selected drug development, the initial trials were single-arm only, with the relevant historical treatment comparators, docetaxel or gemcitabine, having known response rates ranging from 5-15%, and an expected median PFS of 3–5 months (62). The strength of these results led to accelerated approvals for sotorasib and adagrasib, and rush to use a novel targeted therapy in patients.

To some surprise, however, the first randomized trials to report results of G12C inhibitor versus docetaxel in the second line, Codebreak 200 (sotorasib) (63) and Krystal-12 (adagrasib) (64), reported PFS benefits that were real – hazard ratios ~0.6 – but quantitatively small – 5.6/5.5 months versus 4.5/3.8, respectively. As of this writing, both drugs retain their accelerated approvals, but neither have received full or standard FDA approvals based on these results, and neither study has easily interpretable OS results, limited by both sample size and crossover.

While there has been some debate about the clinical design and readouts of these studies, leaving those aside, the simplest explanation is that the effect sizes of these inhibitors are smaller than with other mutant-specific signaling inhibitors in NSCLC. Even the earliest, non-selective EGFR inhibitors had response rates in the equivalent setting (EGFR-selected disease) of at least 50-60% (65, 66). While studies moving G12C inhibitors into the first line might see more efficacy, in like-for-like comparisons of targeted therapies, we do not see such effects. What accounts for this smaller absolute benefit? The answer may be a property of today’s molecules, the signaling structure of RAS itself, or another feature of the tumor – or a combination of all three.

### Are G12Ci responses limited by modest on-target activity?

If our first-generation, GDP-binding G12C inhibitors are simply not potent enough, we should expect lower response rates – or, put another way, moderate primary tumor resistance. Likewise, we would expect advances from molecules with improved medicinal chemistry – whether on-target potency, pharmacokinetic (PK) properties, or both. Hints of this come from dose optimization studies of sotorasib: in a study in patients with lung cancer of 960mg versus 240mg dosing, there was a trend toward higher response rate at the 960mg dose (34% versus 26%), but PFS was disappointingly the same (this is to say nothing of the clinical risk-benefit calculation, which involves toxicity as a variable as well) (67). Notably, drug exposures (Cmax and AUC) were only 1.3 fold higher at 960mg, indicating a saturated PK profile, and limiting the lessons that could be drawn from higher on-target potency. By contrast, in a study in colorectal cancer (CRC) of sotorasib combined with the EGFR monoclonal antibody panitumumab, differences between the dose groups was much more stark: at 960mg the response rate was 26.4% and PFS 5.6 months, versus 5.7% and 3.9 months in the 240mg group (68). It is likely that ERK signaling is relatively harder to suppress in CRC, and so the small change in exposure may lead to non-linear effects, accounting for this difference (69). But there are early hints from other molecules that a similar effect could be hiding in NSCLC. Divarasib, another G12C inhibitor, reported a higher response rate in patients (close to 60%) and, in cellular assays, variably appears 5–20 fold more potent than the first clinical molecules (60). Olomorasib has reported responses after failure of prior G12C inhibitors, and perhaps a slightly different toxicity profile, but otherwise similar response rates to earlier molecules (61). A randomized trial of divarasib versus sotorasib/adagrasib is ongoing, and will directly test the hypothesis that higher on-target potency remains a route to better clinical activity in RAS inhibition (70). We eagerly await results of this study. If potency differences within the same class are a feature of KRAS inhibition, it will be a very different development project than if most molecules have roughly the same activity and major gains will have to be found using complementary mechanisms.

### The landscape of acquired resistance

Could a shorter route to therapeutic resistance explain lower PFS numbers compared to other targets? Evaluations of both baseline (48, 71) and acquired resistance to KRAS inhibition have led to more questions than answers, with results pointing to both a more easily adaptable signaling pathway as well as a more easily adaptable tumor than other oncogene-driven contexts. Predictors of baseline non-responsiveness to G12Ci appear broadly in line with poor predictors of responsiveness to other therapies in NSCLC, such as STK11, KEAP1, and SMARCA4 mutations, with minor differences in effect sizes compared to chemotherapy or immunotherapy (6, 49, 52, 72–75). In studies combining preclinical models, ctDNA-based monitoring, and occasional rebiopsies, multiple mechanisms of resistance were observed, including KRAS amplification (10-20%), acquisition of non-G12C mutations (30-40%), and bypass reactivation of MAPK signaling (~50-60%, including new KRAS mutations) (76–78). A few on-target second-site mutations have been reported, but they are rare, found in about 10-15% of patients. In several cases, putative resistance mechanisms disappeared despite continued disease progression, casting doubt upon their importance (78, 79). This resistance pattern has strikingly more in common with resistance to third-generation EGFR TKIs compared to first-generation (80–84), but has unique features not seen with other TKI subsets. Intriguingly, it appears that novel mechanisms to suppress KRAS signaling, such as the tricomplex molecular glue inhibitors we discuss below, may be effective as salvage therapy in some tumors treated with G12C off inhibitors (85). Regardless of how G12C resistance is modeled, however, human-first studies have led to few obvious rational combinations to either increase response rates or rescue resistance.

### Combination therapies

In the absence of such rational combinations, clinical trials have attempted to port findings from cell line and xenograft models to the clinic to extend the benefit of KRAS inhibitors. To date, most of these strategies have shown limited activity not usually outweighed by increases in toxicity. The most notable success has been the addition of an EGFR monoclonal antibody to KRAS G12C inhibition in colorectal cancer, which was accurately predicted to greatly increase response rates and durations from preclinical models by inhibiting pathway feedback reactivation (86). Other strategies of vertical inhibition (MEK inhibitors (87), SHP-2 inhibitors (88)) or lateral pathway inhibition (HER-family TKIs (89), CDK4/6 inhibitors) have shown at best limited increases in efficacy probably not justifying increased toxicities to patient with NSCLC (90). With these muted results, development programs have fallen back to the old standbys of combining with standard of care treatment: chemotherapy or chemoimmunotherapy, often chosen for pragmatic and standard-of-care reasons, sometimes with limited mechanistic grounding. Much of this is work designed to allow first-line administration of KRAS G12C inhibitors in combination with already established standards of care. Earlier administration of the best drugs has been shown in multiple contexts to have real benefits in NSCLC treatment, and should be pursued. In our view, however, the failure of most modeling-derived combinations should, by contrast, generate a broad rethinking of whether current preclinical modeling strategies accurately recapitulate human toxicities, dose limitations, and resistant cancer biology. Taken together, these findings suggest that, in the clinic, the feasibility of delivering combination regimens at therapeutically effective doses may be at least as important as theoretical pathway synergy, reinforcing the need for combination strategies grounded in both biological rationale and tolerability.

### KRAS inhibitors and immunotherapy

Finally, numerous preclinical studies have identified immune activation in response to KRAS inhibition, leading to predictions that we would observe clinical synergy between KRASi and immune checkpoint blockade (ICB) (91–93). This idea was also encouraged by the covalent mechanism of G12C inhibitors, which can in theory generate unusual neoepitopes for the immune system (94). Given the relevance of immunotherapy to NSCLC treatment in the modern era, such an effect could be transformative, especially for patients not already likely to respond to immunotherapy, i.e., the group with PD-L1 scores <50%. In early clinical results, however, combinations with immunotherapy seem to show the most evidence of any synergistic activity precisely in the opposite group – those with PD-L1 >50% (95, 96). Even here, combination therapy response rates appear mostly additive. While we await data on response durations and a single trial which excludes immunotherapy in the clinical scenario where it is normally offered (97), any potential immune activation from KRAS inhibitors appears to require novel immunotherapy strategies to exploit – not just novel KRAS inhibitors and our current checkpoint inhibitors.

The combination of covalent switch-II G12C inhibitors with immunotherapy has also led to specific toxicities. Hepatitis, observed via elevated transaminases, was noticed during both the sotorasib and adagrasib Phase I studies (58, 98). These were studies ostensibly of monotherapy G12C inhibitor, but when explicit combinations with PD-1 immunotherapy were pursued, observed transaminase elevation rates were even higher (71, 99). This led to a period of discussion about whether such hepatotoxicity was a monotherapy or combination effect, and whether it was unique to sotorasib or a common effect of switch-II covalent G12C inhibitors. Further investigation revealed that the apparent monotherapy toxicity was really a combination toxicity: the vast majority of patients with hepatotoxicity had received checkpoint inhibitor within 6–12 weeks (71, 100). And while hepatitis rates have varied between different G12C inhibitors in combination with checkpoint inhibitors, most have observed at least a continuous low level of this toxicity (96), with the possible exception of non-switch II inhibitors (85). Ultimately, most G12C inhibitors look like they will be combinable with PD-1 therapy, but hepatotoxicity monitoring and management will be an ever-present feature of the combination if it deploys to routine clinical practice.

### Activity in the central nervous system

Many if not most patients with metastatic NSCLC will at some point develop brain metastases. Historically, targeted therapies have had variable CNS activity, and many trials have excluded patients with brain metastases. This habit has been changing, for the better, during the development of G12C inhibitors. Adagrasib reported out CNS activity early in development (101) and several other inhibitors have shown other CNS data (102). In a KRYSTAL-1 cohort of 25 patients with untreated CNS metastases, 19 were radiographically evaluable, and the intracranial ORR was 42% with a PFS of 5.4 months, similar to the numbers for systemic disease control (101). Sotorasib reported evidence of CNS PFS in patients with treated or stable brain metastases, and it likely has similar characteristics as adagrasib, as the intracranial PFS was nearly identical, though in these treated patients (103). But a lack of data on untreated brain metastases with sotorasib has made adagrasib the preferred G12C inhibitor for patients with known CNS disease. This underscores the importance of balanced trial designs that allow for patients with real-world disease characteristics, such as active brain metastases, early in drug development. Olomorasib development included a small (11 patient) dedicated cohort of untreated NSCLC brain metastases with 9 evaluable patients, 6 having an intracranial response (104). Divarasib, while having the highest reported response rate in single-arm studies of G12C inhibitors, has not yet reported out substantial data in untreated brain metastases.

In our view, CNS control is a key component of systemic disease control, and study designs should prioritize ways to enroll patients with CNS disease, even when the exact activity of any individual inhibitor is not known. Radiotherapy techniques are sufficiently successful for local control in the CNS that, more times than not, routine clinical management can allow patients to successfully complete trials and generate useful knowledge. We anticipate that most G12C inhibitors will have similar data from their registrational studies.

### Key future directions in KRAS G12C inhibition

Of the questions above, our own view is that a deep focus on acquired resistance, careful attention to therapeutic index, and new chemical structures are likely to make the benefits from G12C inhibition deeper and more impactful. With most vertical inhibitor combinations showing laboratory benefits that are not recapitulated in humans, new strategies are needed to extend PFS in most patients to a year, two years, or more. Below, we discuss molecular glue inhibitors, whether regular glues or proteolysis-targeting chimeras (PROTACs), which have been used to unlock new ways to target KRAS. These first emerged to target non-G12C states, but are generating drugs in the G12C-allele-specific pipeline as well. Elironrasib, a G12C-selective inhibitor which acts as a molecular glue to abrogate KRAS signaling when it is in the KRAS(ON) state, has shown both response rates in line with prior drugs and, encouragingly, substantial responses after the failure of G12C(OFF) inhibition (85). Dual on-and-off-state inhibitors that are not molecular glues, such as BBO-8520 and FMC-376, have also entered clinical trials, with results eagerly awaited (105–107). One potential future for G12C(OFF) inhibition is thus as a single tool in a toolkit of RAS-targeting molecules, combined with pan-(K)RAS state inhibitors and other combinations that specifically address or abrogate known resistance mechanisms. This would be a similar approach to amivantamab eliminating MET-mediated resistance to EGFR inhibition (108). ctDNA-guided approaches, where emergent resistant clones might be detected before clinical relapse and treated, could hold particular promise (79, 109). The next several years will hopefully see not just progress to first-line use, but novel biological strategies that help patients live noticeably longer.

## Targeting non-G12C RAS alleles

### Chemical techniques for novel RAS inhibition beyond inhibitors

Only a minority of the total RAS-mutated cancer burden contains G12C mutations: G12D, G12V, and G12A are an order of magnitude more common in PDAC and CRC. Even in NSCLC, G12C accounts for less than half the number of KRAS-mutated cases and a smaller number of the RAS-mutated whole (counting HRAS and NRAS). This has spurred a substantial race to generalize G12C inhibitors and to create either allele-specific or pan-(K)RAS inhibitors.

Before discussing a list of in development KRAS-targeting agents, it is worth a return to our classification schema in **Figure 1**. Each KRAS mutation – and the possibility of pan-(K)RAS inhibition – presents novel pharmacologic problems. Consider the G12C inhibitors: allele-specific, covalent, RAS(GDP)-bound inhibitors. These relied on an accessible covalent difference from wildtype protein, a shallow but present pocket, and substantial GTP-GDP state cycling. None of these features are present for a chemist attempting to inhibit, for example, KRAS G12D. G12D mutations have stronger oncogenic activity in models than G12C mutation (110, 111) likely due to reduced cycling, so the GDP-bound state is rarely accessible (18). The aspartate side chain that replaces glycine has the potential for covalent or hydrogen binding, but the kinetics are slower and the strength weaker than with cysteine, limiting the ability to rely on this screening strategy. By increasing non-covalent contact points on the protein, one could end up with an inhibitor of all KRAS(GDP) activity, rather than a specific allele. If trying to design a pan-KRAS inhibitor, this may be the goal – or the resulting inhibition of wildtype KRAS may result in a loss of therapeutic index. These challenges have prompted both derivatization of KRAS G12C inhibitors along these avenues as well as the exploration of unrelated techniques to make small molecules that could inhibit RAS.

Just as a rational use of covalency cracked open the decades-old problem of drugging RAS, these novel techniques were added to the drug discovery repertoire at just the right time to target alleles beyond G12C. The most important of these are the use of drugs making use of induced proximity, or so called multicomplex inhibitors: molecular glues and the subset of glues which are proteolysis-targeting chimeras (PROTACS). Molecular glues are small molecules which stabilize a protein-protein interaction (PPI) which would not otherwise occur, or occur only weakly, generating a new multimember complex (112). If the goal is to disable one member of the complex, this can be achieved either by steric occlusion of normal binding partners, or, alternatively, by bringing an enzymatic activity into close proximity such that it modifies the target protein, rendering it permanently incapable or even destroyed. Both modular and non-modular glues exist; the bifunctional, modular glues which recruit an E3 ligase in contact with a target protein, thereby tagging it with ubiquitin for degradation, are PROTACs (113, 114).

Molecular glues have several unique properties compared to direct inhibitors. First, binary binding activity to a target protein may be modest or non-existent, and potency emerges only from induced cooperativity in the ternary complex. This means both the target and the partner must always be present in the cell of interest, and receptive to complex formation. Second, by exploiting composite surfaces formed only in the ternary complex, molecular glues expand the druggable proteome to proteins lacking conventional or “deep” pockets. Third, degraders and some other glues have event-driven pharmacology: once ubiquitination and degradation occur, the small molecule can turn over and generate another ternary complex and participate in additional degradation. All of these features have made molecular glues a key component of the approach to the “undruggable” proteome, including RAS alleles beyond G12C.

### From G12C to G12D switch-II inhibitors

The first drugs to enter the clinic after G12C inhibitors, however, were not glues and degraders but derivatives of G12C inhibitors derivatized to meet the challenges of binding other alleles, usually in the familiar Switch II pocket.

G12D is the most common KRAS allele pan-cancer. As such, this made it a natural first target to extend KRAS targeting. While KRAS G12D is about as common in NSCLC as ALK rearrangements or EGFR exon20 insertions, there is an order of magnitude more patients with KRAS G12D PDAC, where it also makes up a more substantial portion of total cases (near 50%) (10). This has meant that, in a near-flip of the scenario with G12C targeting, PDAC enrollment has dominated early studies of G12D-targeting molecules. But there is a second implication of this epidemiology: a rarer disease entity sometimes leads to the superficially counterintuitive scenario where pathways to regulatory approval are shorter. For example, despite enrolling an order of magnitude more lung than colorectal patients, the first full approval for a G12C inhibitor was in colorectal cancer, not lung cancer, because the FDA recognizes differing feasibility of trial designs in different contexts (68, 115). We suspect we will see a similar phenomenon with G12D inhibitors. The population is small enough in lung cancer that accelerated approvals based on single arm studies, or smaller randomized studies in later-line settings, may suffice for registration.

MRTX1133, the first reported G12D inhibitor, is a noncovalent, allele-selective, dual-state, reversible inhibitor which acts through high affinity interactions that overcome the need for covalent bonding (116, 117). The inhibitor takes advantage of potent hydrogen bonding between an amine and the aspartate side chain to keep mutant KRAS^G12D^ in its GDP-bound, inactive form, and conformational changes in GTP-bound form impair effector binding. After initially demonstrating promise in murine xenograft tumor models, MRTX1133 subsequently progressed to Phase 1 clinical trial (NCT05737706) beginning in March 2023 (118, 119). Unfortunately, this trial is no longer enrolling patients, perhaps due to inadequate pharmacokinetic properties for routine clinical administration. As a proof-of-principle for G12D targeting, and in lab studies, MRTX1133 remains an important milestone; but it is no longer under clinical development.

Several other G12D inhibitors with similar chemical concept to MRTX1133 have been reported, and some have showed more encouraging clinical data. All the data are early – from Phase 1/1b studies. HRS-4642 reported similar preclinical data to MRTX1133 (120) but more encouraging clinical response rates of 20-25% in PDAC and 30% in NSCLC (121) in an escalation/expansion study conducted in China. INCB161734 also showed a ~30% ORR (8/27 patients), though only among non-NSCLC patients (122). GFH375/VS-7375 reported a near-identical ORR (6/22, 27.3%) including 7 patients with NSCLC early in development. Last year, with updated data from a China-only cohort, the sponsor reported a 68% confirmed response rate at the recommended phase 2 dose, and 57% across all dose levels, in 28 NSCLC patients (123). If recapitulated in larger international cohorts, these numbers compare favorably to response rates seen in mature TKIs in NSCLC. A half-dozen other molecules with reportedly similar structure – at least – are in clinical trials but yet to report results, such as GDC-7035. Early toxicity data suggests a similar profile to the G12C inhibitors, with gastrointestinal side effects predominating, but few MTDs reached and generally tolerable reactions – so far as early phase data can tell us.

KRAS G12D has also been the first locus of degrader-therapy development for NSCLC. The first induced-proximity drug to present a novel mechanism against KRAS is setidegrasib (ASP3082), a G12D selective VHL PROTAC (124). The molecule was optimized for ternary complex formation and reported inhibition of MAPK signaling in the 10-30nM range. In large, international clinical trials of patients with refractory pancreatic and lung cancers (second, third, and fourth line), at recommended phase II doses, response rates in the 30% range with pancreatic cancer and 40% with NSCLC were observed (125, 126). As with many PROTACs, pharmacokinetics might be a challenge: the drug was administered via weekly infusion. But importantly, no maximum tolerated dose was noted, consistent with high selectivity for just KRAS G12D, and as proof of principle for what might be achieved with ternary complex-induced, allele-selective pharmacology. Setidegrasib is entering Phase III studies in lung and pancreatic cancer, the first allele-specific non-G12C therapy to do so.

Whether PROTAC or inhibitor technologies have an advantage here will be a recurring theme over the next several years of KRAS drug development. Several additional PROTACs, such as ARV-806 (127), have just started trials (128), and will provide further data on this mechanism. G12D-targeting drugs which are structurally distinct from the switch-II inhibitors and PROTACs are discussed below in the section on tricomplex inhibitors.

### Pan-KRAS or multi-allele KRAS inhibitors

The ability of inhibitors to bind to G12D raised the possibility of designing inhibitors that bind to the Switch II pocket of KRAS with some affinity, regardless of mutant allele, or that may include inhibition of WT KRAS. These are often termed pan-KRAS inhibitors, or multi-allele selective KRAS inhibitors. They generally are chemical and mechanistic successors of the G12C(GDP) binding molecules which, similar to G12D specific inhibitors, bind to both the on/off states, prevent cycling to the on state and disrupt downstream effector binding, and have shown preclinical inhibition of one or more KRAS mutations. It is important to distinguish these from pan-RAS molecules and allele-specific molecules, and that is where they fit on the schema of **Figure 2**.

Many of these molecules are, as of today, in early dose-escalation/dose-finding clinical trials (**Table 1**), perhaps with some reported responses – but it is early in their development cycle. BBO-11818 is an archetype of this class, with preference for G12V and G12D alleles, but 1000-fold lower potency against cell lines with NRAS or BRAF mutations compared to KRAS mutations (129). LY4066434 is a pan-KRAS inhibitor which shows activity in preclinical models against most KRAS alleles, including WT KRAS, but excepting G12R (130). It is now in trials enrolling patients with that exception (131). ALTA3263, with preference for G12C/D/V and low nanomolar potency also recently entered Phase 1 studies (132). PF-07934040, AMG-410, JAB-23425, and others all have similar reported mechanisms and similar clinical development statuses. As of today, some of these remain in development, while clinical studies have halted in others, sometimes with limited public disclosures. Earlier generations of similar molecules, such as BI-2865 or BI-3706674, lacked binding to the on state of the protein, but shared the pan-KRAS inhibition and strong preclinical results (133, 134). Ongoing clinical studies are testing this molecule only in a very specific disease state (upper GI cancers with KRAS amplification), suggesting preclinical potency does not translate for off-only inhibitors into broad clinical activities – or that it has just not yet been validated in this context.

**Table 1:** Active direct RAS-targeting molecules in clinical trials in NSCLC.

Drug	Target	Mechanism	Clinical trial NCT number(s)	Clinical trial name(s)
G12C Inhibitors				
Sotorasib (AMG 510; Lumakras/Lumykras)	KRAS G12C	Inhibitor (covalent; GDP/Switch-II pocket)	NCT03600883 NCT04185883 NCT04303780 NCT05198934	CodeBreaK 100 CodeBreaK 101 CodeBreaK 200 CodeBreaK 300
Adagrasib (MRTX849; Krazati)	KRAS G12C	Inhibitor (covalent; GDP/Switch-II pocket)	NCT03785249 NCT04613596 NCT04793958 NCT04685135	KRYSTAL-1 KRYSTAL-7 KRYSTAL-10 KRYSTAL-12
Divarasib (GDC-6036)	KRAS G12C	Inhibitor (covalent; GDP/Switch-II pocket)	NCT04449874 NCT04929223 NCT06497556 NCT06793215	GDC-6036-001 (FIH) INTRINSIC (umbrella) Krascendo 1 Krascendo 2
Calderasib (MK-1084)	KRAS G12C	Inhibitor (covalent; GDP/Switch-II pocket)	NCT05067283 NCT06345729 NCT07190248 NCT06997497	KANDLELIT-001 KANDLELIT-004 KANDLELIT-007 KANDLELIT-012
Olomorasib (LY3537982)	KRAS G12C	Inhibitor (covalent; GDP/Switch-II pocket)	NCT04956640 NCT06119581	LOXO-RAS-20001 (phase 1) SUNRAY-01
Glecirasib (JAB-21822)	KRAS G12C	Inhibitor (covalent; GDP/Switch-II pocket)	NCT05009329 NCT05002270	JAB-21822-101 (phase 1) JAB-21822-1001 (phase 1/2)
Garsorasib (D-1553)	KRAS G12C	Inhibitor (covalent; GDP/Switch-II pocket)	NCT04585035 NCT05383898	D-1553 phase 1/2 (solid tumors) D1553-102 (NSCLC)
Elisrasib (D3S-001)	KRAS G12C	Inhibitor (covalent; GDP/Switch-II pocket)	NCT05410145	D3S-001 phase 1/2 (FIH)
Fulzerasib (IBI351/GFH925; Dupert®)	KRAS G12C	Inhibitor (covalent; GDP/Switch-II pocket)	NCT05005234 NCT05497336 NCT05756153 NCT05504278 NCT06936644	GFH925/IBI351 monotherapy (phase 1/2; registrational) GFH925/IBI351 monotherapy (phase 1) KROCUS (GFH925 + cetuximab) IBI351 combinations (phase Ib/III) IBI351 + ivonescimab (phase II)
Elironrasib (RMC-6291)	KRAS G12C (ON)	Molecular glue / tri-complex inhibitor (RAS(ON))	NCT05462717 NCT06128551 NCT06162221 NCT07397338	RMC-6291-001 (phase 1/1b) Elironrasib + daraxonrasib (combo) NSCLC platform study of RAS(ON) inhibitors RAS(ON) inhibitors + ivonescimab (combo)
HRS-7058	KRAS G12C	Inhibitor (small-molecule)	NCT06383871 NCT06915142	HRS-7058-? (phase 1) HRS-7058-201 (phase 2 combinations)
FMC-376	KRAS G12C (dual ON+OFF reported)	Inhibitor (dual-state; small-molecule)	NCT06244771	PROSPER (phase 1/2)
G12D Inhibitors				
MRTX1133	KRAS G12D	Inhibitor (non-covalent; KRAS G12D)	NCT05737706	MRTX1133-001 (phase 1/2; no public acronym)
Zoldonrasib (RMC-9805)	KRAS G12D (ON)	Molecular glue / tri-complex inhibitor (RAS(ON))	NCT06040541	RMC-9805-001 (phase 1/1b)
ASP3082	KRAS G12D	PROTAC / targeted degrader	NCT05382559	ASP3082 first-in-human (phase 1)
ARV-806	KRAS G12D	PROTAC degrader	NCT07023731	ARV-806 phase 1 (KRAS G12D-mutant advanced cancers)
HRS-4642	KRAS G12D	Inhibitor (small-molecule)	NCT05533463 NCT06385678	HRS-4642 phase 1 HRS-4642 + immunotherapy combo study
VS-7375 (also known as GFH375; Verastem/GenFleet)	KRAS G12D (ON/OFF)	Inhibitor (non-covalent; ON/OFF)	NCT07020221 NCT06500676	VS-7375-101 (phase 1/2a) GFH375 phase 1/2 (China)
KQB548 (BAY 3771249)	KRAS G12D	Inhibitor (small-molecule)	NCT07207707	KQB548-101 (phase 1a)
QTX3034	KRAS G12D (trial inclusion); multi-KRAS profile reported	Inhibitor (small-molecule)	NCT06227377	QTX3034 phase 1 (advanced solid tumors with KRAS G12D)
QTX3046	KRAS G12D	Inhibitor (small-molecule)	NCT06428500	QTX3046 phase 1 (advanced solid tumors with KRAS G12D)
INCB161734	KRAS G12D	Inhibitor (small-molecule)	NCT06179160	INCB161734 phase 1 (advanced solid tumors with KRAS G12D)
HS-10529	KRAS G12D	Inhibitor (small-molecule)	NCT06963398	HS-10529 phase 1 (advanced solid tumors with KRAS G12D)
ASP4396	KRAS G12D	Inhibitor (small-molecule; Astellas program)	NCT06364696	ASP4396 phase 1
pan-KRAS inhibitors				
BGB-53038	Pan-KRAS (mutations or amplification)	Inhibitor (small-molecule)	NCT06585488	BGB-53038 FIH (phase 1a/1b)
LY4066434	Pan-KRAS	Inhibitor (small-molecule)	NCT06607185	LY4066434 phase 1 (advanced solid tumors with KRAS mutations)
ERAS-4001	Pan-KRAS (KRAS-mutant tumors)	Inhibitor (small-molecule)	NCT07021898	BOREALIS-1 (phase 1)
BBO-11818	Pan-KRAS	Inhibitor (small-molecule)	NCT06917079	BBO-11818 phase 1
PT0511	Pan-KRAS (multiple KRAS variants; KRAS mutated/amplified)	PROTAC / degrader (pan-KRAS degrader)	NCT07300150	PT0511 phase 1
ASP5834	Certain KRAS mutations	Inhibitor (small-molecule)	NCT07094204	ASP5834-CL-1001 (phase 1)
pan-RAS inhibitors				
Daraxonrasib (RMC-6236)	Pan-RAS (ON) (mutant & WT RAS, per protocol)	Molecular glue / tri-complex inhibitor (RAS(ON))	NCT05379985 NCT06625320 NCT06881784 NCT07252232 NCT06162221 NCT07397338 NCT06128551	RMC-6236-001 (phase 1) RASolute 302 (phase 3) RASolve 301 (phase 3) RASolute 304 (phase 3) Platform study of RAS(ON) inhibitors in NSCLC RAS(ON) inhibitors + ivonescimab Elironrasib + daraxonrasib (combo)
ERAS-0015	Pan-RAS (mutant RAS tumors)	Molecular glue (pan-RAS)	NCT06983743	AURORAS-1 (phase 1)
GFH276	Pan-RAS (ON) (WT & mutant RAS)	Molecular glue (pan-RAS)	NCT07198321	GFH276 phase I/II
YL-17231 (TEB-17231)	Pan-RAS (KRAS/NRAS/HRAS mutations)	Inhibitor (pan-RAS)	NCT06096974	YL-17231 phase 1/2
AN9025	Pan-RAS (ON)	Inhibitor / tri-complex (pan-RAS(ON))	NCT07252479	AN9025 phase 1 (dose escalation/expansion)
Allele-specific, non-G12C/D				
RMC-5127	KRAS G12V (ON)	Molecular glue / tri-complex inhibitor (RAS(ON))	NCT07349537	RMC-5127-001 (phase 1; monotherapy ± combinations)
KQB365	KRAS G12S and KRAS G12C	Inhibitor (KRAS mutant inhibitor)	NCT06720987	KQB365 phase 1/1b (monotherapy ± combinations)

### Pan-RAS inhibitors

At the same time as setidegrasib entered the clinic as a PROTAC targeting G12D, another set of molecular glue molecules without E3 ligase activity entered the clinic to target a variety of different RAS states. These are “tricomplex inhibitors,” which act by a multi-step pharmacology that binds to the RAS(ON) complex and disrupts downstream signaling. The first step of the mechanism is binding to a ubiquitous cellular protein, in this case, cyclophilin A (CYPA), which normally has no affinity for RAS. In the presence of drug, however, cyclophilin is “glued” to RAS and prevents RAF and other effectors from binding. Thus, even while on, RAS cannot signal. (This happens to also be the mechanism of cyclosporine – the calcineurin inhibitor – which binds to cyclophilin A and then to calcineurin. The molecule is simply tuned for RAS instead.) The implications of this mechanism are several, including a requirement for CYPA binding and availability; tunability to specific allele states; and rapid inactivation of RAS signaling compared with stabilizing an (OFF) state. Without the protein turnover of PROTACs, this is also not event-driven pharmacology: one RAS molecule requires a cognate CYPA molecule stabilized on its surface, interfering with activity.

The first-in-class of these drugs was daraxonrasib (RMC-6236), a RAS(ON), pan-RAS (binding all K-, H-, and N-RAS alleles), noncovalent molecular glue. In preclinical studies with a tool compound, daraxonrasib demonstrated strong activity across multiple RAS alleles, including against G12C(GDP)-resistant tumors (135). Daraxonrasib also inhibits wildtype RAS with some strength, and one surprise of the activity of daraxonrasib was that it appeared tolerable in mice. Subsequent work revealed the basis of the therapeutic index was in differential responses to drug of RAS-activated tumor versus normal tissue (136), and that tricomplex inhibitors could even restore normal GTP hydrolysis in normally incompetent mutants to aid in their antitumor activity (137).

On the basis of these preclinical data, daraxonrasib entered clinical trials for NSCLC and PDAC patients harboring essentially any pathogenic KRAS mutation, including G12 mutations and Q61 mutations. Separate cohorts also evaluated the drug in patients with NRAS or HRAS mutations, and in diverse tumor types outside NSCLC and PDAC. A response rate of near 30% was observed in later-line pancreatic cancer, and up to 40% in later line lung cancer, with responses seen across essentially all KRAS mutations (138–141). Responses often lasted months-to-years, with an average duration of response of at least 10 months. As with many newer targeted therapies, no formal DLTs were reached, but dose optimization studies led to a recommended dose of 200mg daily in NSCLC and 300mg daily in PDAC. Intriguingly, serum pharmacokinetics shows higher median concentrations in NSCLC patients; it is also possible that PDAC has intrinsically higher therapeutic resistance compared to NSCLC. Based on these results (141), daraxonrasib started registrational Phase III trials in PDAC against investigators' choice second line chemotherapy (142), and in NSCLC against second line docetaxel (143). Results of the former have been announced, but not yet presented: daraxonrasib doubled overall survival, according to a sponsor release of limited data. While the full dataset is not yet released, the headline number indicates the potential for transformative outcomes from the right RAS therapy and the right disease state. Enrollment for definitive studies of daraxonrasib in NSCLC may take slightly longer. Nonetheless, we anticipate that daraxonrasib will complete these randomized Phase III studies before any other non-G12C RAS inhibitor in NSCLC, and first-line safety studies (combining with chemotherapy and immunotherapy) are already underway.

There are several unique clinical features of daraxonrasib. The first is its wide potential application to essentially all KRAS mutated NSCLC, as well as its inhibition of N- and H-RAS (“pan-RAS” as opposed to “pan-KRAS”). Next is the implications of wildtype RAS inhibition, manifested by its toxicity profile. The most notable toxicities with daraxonrasib were mucosal – especially rash. While generally well-tolerated and leading to few dose reductions in the early phase trials, some form of rash occurred in up to 90% of patients at the higher doses, and G2 or G3 rash could occur in 20-30%. This generally appeared similar to an EGFR rash in distribution and morphology (144). Stomatitis and other mucosal toxicity was also observed, whereas gastrointestinal toxicities were mild and hepatotoxicity was almost nonexistent. This is a unique pattern compared to both the G12C, G12D, and perhaps the pan-KRAS inhibitors as well. The mechanism of action of rash is not fully understood but is likely to result from the fact that there is no compensatory activity of HRAS or NRAS (also inhibited by daraxonrasib) in normal skin tissue while drug is active. In the future, multiple strategies may be needed to combat this bothersome side effect. Still, daraxonrasib has received breakthrough designation from the FDA, and trial results are eagerly anticipated in the coming years.

There are other RAS inhibitors in development with the tricomplex mechanism. ERAS-4001 is a novel pan-RAS inhibitor, which recently began recruiting for Phase I clinical trial (NCT07021898) in August 2025 (145, 146). Beigene, Genentech, and others have publicly announced pipeline molecules which have similar properties. Some have claimed increased potency compared to daraxonrasib. But the key question, as usual, will not be potency – but rather therapeutic index. With pan-RAS molecules, it is not clear that these two features will proceed linearly together.

### Allele-specific tricomplex inhibitors

From proof-of-principle with daraxonrasib, several allele-specific (rather than pan-RAS) RAS(ON) inhibitors are now also in clinical trials. The first of these was elironrasib (RMC-6291), a G12C-specific, covalent, RAS(ON) inhibitor (147). It was initially tested in patients who had either received or not received a prior G12C(OFF) inhibitor, where in dose escalation it reported a tolerable safety profile, little hepatitis, and a response rate of around 60% (85, 148). Consistent with preclinical data showing elironrasib could elicit responses in patients resistant to G12C(OFF) inhibitors (149), a cohort which specifically enrolled patients who had previously progressed on these had a response rate of 42% to elironrasib monotherapy (N = 24). Another trial enrolled patients with elironrasib and daraxonrasib administered together (NCT06128551), for which data have been shown publicly, showing a 40-60% response rate, but not yet presented or published. In the initial work describing resistance to G12C(OFF) inhibitors, secondary KRAS mutations were common, and it is an appealing hypothesis that concurrent pan-RAS inhibition would abrogate this resistance mechanism and lead to longer responses (76). Elironrasib, like the other G12C inhibitors, has completed some initial safety studies with pembrolizumab and with chemotherapy. Whether an allele-specific RAS(ON) inhibitor will show stronger efficacy upfront compared to any RAS(OFF) inhibitor – or whether they might be well-used in the resistant setting – will be an important question over the coming years.

The next step in the use of tricomplex inhibitors was to build allele-specific molecules against novel alleles. Zoldonrasib (RMC-9805) is a G12D-specific tricomplex inhibitor with similar structure to elironrasib and daraxonrasib. As with all G12D inhibitors, initial dose finding studies enrolled mostly patients with PDAC, where the ORR was 30%, similar to daraxonrasib, if not slightly higher (150). In a smaller cohort of NSCLC patients, zoldonrasib had a remarkable response rate of 60% (151). In an updated analysis of 27 patients who had not received docetaxel and were efficacy evaluable, the response rate was 52%. Notably, the safety profile of elironrasib and zoldonrasib appear more similar to one another – with few high-grade toxicities – than to daraxonrasib. Plausibly, allele-specific inhibitors may generate the best balance between toxicity and response, with pan-RAS inhibitors greatest use in rare alleles, as an adjunct, and in combination therapies. Whether or not this proves to be true, a surfeit of allele-specific molecules – an entire toolkit – could offer a bevy of regimens to explore for RAS mutant tumors. Zoldonrasib also received breakthrough therapy designation from the FDA and is under investigation in a just-completed, registrational, single-arm cohort (NCT06162221) and a planned Phase III first-line study.

The final clinical tricomplex inhibitor is RMC-5127, the first G12V-selective RAS(ON) inhibitor (152). This has just started clinical trials, following a similar path as the prior three molecules. Multiple other allele-specific RAS inhibitors have been announced in drug pipelines, but information is relatively scarce. The “tunable” nature of platforms such as the tricomplex inhibitors is one of the advantages of modular chemical techniques, shared with PROTACS. The ability to rapidly generate these molecules is also likely to mean that clinical trial eligibility and enrollment will become our biggest bottleneck to advancing RAS-directed therapies, rather than chemical discovery (153).

### Allele specific versus pan-RAS versus KRAS inhibition

Is pan-RAS inhibition superior or inferior to mutant-selective inhibition? This will be a key question as more RAS-targeting molecules enter the clinic, and clinicians and researchers will be faced with opportunities to use an allele-specific inhibitor, a pan-KRAS or pan-RAS inhibitor, or combinations of all of the above. For each of the more advanced molecules – setidegrasib, zoldonrasib, elironrasib, and daraxonrasib – multiple clinical trials are testing the molecules alone, in combination with each other, or in combinations with chemotherapies and immunotherapies.

In our view, the most likely rule which will determine the outcome of these trials is: there will be no one rule. For combinations with chemotherapy, it is likely that tolerability will predominate as a concern. “Synergy,” the often-sought, rarely-observed phenomenon in clinical cancer therapy (154, 155), may play a role in determining which combination therapies are superior. But equally plausibly, whether two therapies can simply be given safely and efficiently, simultaneously, may drive outcomes. And since these molecules are all in early stages of development, any synergistic activity may take a long time to reveal itself. In EGFR mutant lung cancer, for example, the addition of chemotherapy upfront to EGFR TKIs seems to confer long-term progression free benefit – but adds relatively little to response rates (156). In publicly-reported but unpresented data of patients with PDAC, daraxonrasib in the 1L led to a 40% response rate, but the addition of gemcitabine and nab-paclitaxel – which normally has response rates of around 20% in this setting – only added 7% to daraxonrasib monotherapy, for a combination response rate of 47%. Another important consideration will be control of CNS disease. Combination chemotherapy has occasionally seemed to lead to better brain control in agents which are already active in the brain. We do not yet know CNS activity for most of these newer RAS-targeting agents, and we anticipate these data.

We expect the next several years will be devoted to figuring out what the composition of our RAS toolkit will be – and then how to use the tools together. Combinations of pan-(K)RAS inhibitors with allele-specific inhibitors, with or without standard of care therapies, is one important avenue of exploration. With a lesson from the G12C inhibitors, we are skeptical of vertical or other combinations, especially with overlapping toxicities – such as an EGFR inhibitor plus daraxonrasib, both of which induce rash. Some early resistance data has been presented, and will be key for future studies (157, 158). In the end, it is better to have more tools than fewer, but simply trying all the tools in all the combinations is likely to lead to many abandoned avenues.

### Paths to approval alone and in combination

The key question faced by many of these therapies over the next few years is how to progress toward FDA approval: which setting and when? The first expected results will be of Phase III, randomized studies of daraxonrasib and setidegrasib versus standard of care second line chemotherapy (almost always docetaxel), similar to the registration pathway for G12C inhibitors. Factoring in likely attrition of response rates from Phase I to Phase III studies, the lower bound for efficacy of these molecules appears broadly similar to the G12C inhibitors – just now able to target different mutation-selected populations. For single mutation inhibitors other than G12C, it is possible that single-arm studies, with sufficient response rate and duration data, might allow for more rapid accelerated approval, as has previously been the norm for NSCLC therapies with mutation-targeting and similar population sizes, such as MET or ALK inhibitors. Confirmatory trials might then move to first line or even combination settings right away.

The first-line standard of care for most patients with KRAS mutations remains immunotherapy plus or minus chemotherapy (159, 160). In some cases – mostly in patients responsive to PD-1-axis immunotherapy – this is an excellent option that can lead to long-term disease control. This includes patients with KRAS G12C mutations, who commonly have a history of smoking, which is one of the better predictors of immunotherapy responsiveness (161). But smoking history is varied among other mutation subtypes. In a retrospective epidemiologic study of patients with G12D NSCLC, for example, roughly 22% were found in never smokers, versus 5% of G12C NSCLCs (33). Self-reported smoking history – or secondhand exposure – may also not correlate with genomic signatures of smoking in all cases, and non-smoking exposures can generate similar immunotherapy-responsive biology, at least in principle (162). Analyses of responsiveness to novel KRAS inhibitors by reported smoking status and by concurrent smoking signatures will be an important barometer for assessing whether these patients should receive immunotherapy and chemotherapy or targeted monotherapies preferentially. We’ve worked out the case for EGFR mutations; now the question is working it out for the more complex landscape of KRAS mutations. First line studies combining KRAS inhibitor with chemoimmunotherapy against chemoimmunotherapy alone will almost certainly be completed before, or along with, availability of such data. But the truest test for novel KRAS inhibitors will be whether they can allow us to rethink our whole treatment paradigm, rather than merely build on top of it.

Practically speaking, registrational first-line trials in combination with chemotherapy and immunotherapy are already planned or about to enroll for daraxonrasib and zoldonrasib. We believe that integration of newer biomarkers – those closer to tumor biology – will take on outsized importance for novel combinations, such as RAS-RAS combinations. ctDNA-based resistance monitoring (78, 79) might allow for new trials designed to intervene on ctDNA changes before radiographic changes become apparent; for example as non-G12C mutations emerge, adding a pan-RAS inhibitor on to suppress them. Breast cancer is ahead of lung cancer with such designs (109). If smoking-related signatures allow for dichotomized outcomes, these too could inform first line study designs or rearrange therapy ordering, and studies should ideally be powered to test such rational, pre-specified subgroups based on disease biology. We will learn much from the current crop of G12C inhibitor first-line studies, but G12C mutations and G12D mutations and G12V mutations may have less in common than first glance.

Other drugs are constantly being developed in NSCLC – anti-body drug conjugates (ADCs), or novel targeted therapies for SMARCA4 mutated and MTAP-deleted tumors, for example – and many of the addressable populations may have substantive overlap with patients with KRAS mutations. Such “novel-novel” combination therapies may have biological rationale in many cases. A collaboration between Tango Therapeutics and Revolution Medicines is already testing the combination of RAS inhibition and MTAP-targeting PRMT5 inhibitors in MTAP-deleted, KRAS-mutated tumors (NCT06922591); MTAP-directed therapies tend to act slowly and RAS inhibitors quickly, making them natural combination partners on clinical response kinetics alone (163–165). Whether they will improve outcomes based on non-overlapping biology is a separate question. ADC combinations with targeted therapy have been common in EGFR-mutated disease and are likely to make their way to KRAS-mutated disease as well. The surface biology of KRAS mutated disease is not well known, either *de novo*, or in response to KRAS inhibition. Finally, a combination of these modalities not in different drugs – but in one therapy – could be coming. ADCs targeted against CEA, but delivering a pan-RAS inhibitor, have entered clinical trials for GI cancers (166). While far too early to know what will come of this strategy, it is a novel concept to generate greater therapeutic index and deserves testing.

## Future strategies and directions for KRAS targeting

Herding – the tendency for multiple drug development programs to all go after the same target, at once – is common in biomedical drug development, and often unproductive (167). Yet the field of KRAS targeting has never looked healthier: full of novel chemical concepts, competing mechanisms, and many new ideas. The next few years will see readouts of first line G12C inhibitor trials, evidence about whether on target G12C potency differences can make a difference to long-term disease control, and new evidence about synergistic activity with immunotherapy. Non-G12C pan-RAS inhibitors are likely to finish enrolling definitive later-line studies, allele-specific tricomplex inhibitors may move toward registrational cohorts, and a surfeit of molecules now being explored in dose escalation will report more complete datasets that will include reasonably-sized early efficacy readouts. It will be busy.

The barometer of success will be whether these advances transfer to improved patient outcomes. What would this look like? In practical terms, a meaningful benchmark for success in KRAS-directed therapy would be to approach the depth and durability of benefit observed with other oncogene-directed therapies in NSCLC, such as EGFR or ALK inhibitors, where response rates routinely exceed 60–70% and median progression-free survival extends beyond one year. Whether this level of efficacy is achievable in KRAS-mutant disease remains uncertain, but it provides a useful clinical comparator against which emerging therapies can be evaluated. At a minimum, durable disease control beyond that achievable with chemoimmunotherapy, particularly in molecularly defined subgroups, would represent a substantive advance. Repeated re-targeting of the pathway, through combination allele-specific, pan-RAS, and other mechanism-based approaches, would be another.

Whether or not we achieve that success, the last several years of RAS-directed NSCLC therapy development have held remarkable lessons for future NSCLC drug development broadly.

The first is that chemical and pharmacological innovation is key. Multiple molecules competing in the same space, derived from similar scaffolds, may rearrange the chairs based on clever clinical trial design. But more often than not, mechanistic or chemical innovation along the right axis will be the most important. In some cases, this may be increased potency, as with G12C inhibitors, and perhaps other allele-specific molecules. But with others – such as pan-RAS inhibition – the real key question will be increasing therapeutic index, or better sparing wildtype RAS. For the same reason, combination therapies on the same target, or hitting compensatory, unmutated signaling nodes, tend to look much more tolerable in lab studies than they often end up being in humans. There are exceptions, but few.

The second is that regulatory schemas may need elaboration to support such diverse precision medicine drug development in a world in which small-molecule productivity is no longer the major bottleneck. More patients, on more trials, will be required to generate all this information. Sponsors may have to realize that they may not be able to enroll multiple similar first-line therapy trials at the same time, especially when another drug in the same class is already approved and there are limited if no differences. Studies guided by novel markers, or in late line settings with clever biology, can still move the field in new directions. Diverse contexts and designs help generate better knowledge bases and lead to new avenues of exploration – and drug approval.

Finally, tumor biology remains the king. Clinical and statistical considerations may drive much of trial design. But the lesson of EGFR inhibition – which was initially tested in all-comers, only later to discover it was only mutated patients who benefited – is that sufficiently strong biology can overcome even weak statistics. There remains no substitute for testing RAS inhibitors in human patients with correlative studies at resistance that lead directly to testable hypotheses, and combination regimens which are based on systematic investigation rather than approved therapeutic availability. A surfeit of interest means that RAS-targeted therapies could lead this way.

After nearly fifty years of failure, it is remarkable how much has been achieved in just over a decade, and we remain hopeful that truly impactful RAS inhibition will result in lasting benefits for the too many lung cancer patients who still suffer from RAS-driven cancers each year.
